# Spinal Neuromodulation for Respiratory Rehabilitation in Patients with Post-Acute COVID-19 Syndrome

**DOI:** 10.3390/life14111518

**Published:** 2024-11-20

**Authors:** Alexander Ovechkin, Tatiana Moshonkina, Natalia Shamantseva, Vsevolod Lyakhovetskii, Aastha Suthar, Niraj Tharu, Alex Ng, Yury Gerasimenko

**Affiliations:** 1 Kentucky Spinal Cord Injury Research Center, University of Louisville, Louisville, KY 40202, USA; aastha.suthar@louisville.edu (A.S.); niraj.tharu@louisville.edu (N.T.); yury.gerasimenko@louisville.edu (Y.G.); 2 Department of Neurological Surgery, University of Louisville, Louisville, KY 40202, USA; 3 Pavlov Institute of Physiology Russian Academy of Sciences, 199034 St. Petersburg, Russia; moshonkina@infran.ru (T.M.); shandibinan@infran.ru (N.S.); lyakhovetskiiva@infran.ru (V.L.); 4 Department of Medicine, Division of Pulmonary, Critical Care and Sleep Disorders Medicine, University of Louisville, Louisville, KY 40202, USA; alex.ng@louisville.edu; 5 Department of Physiology, University of Louisville, Louisville, KY 40202, USA

**Keywords:** neuromodulation, spinal cord stimulation, respiration, rehabilitation, COVID-19

## Abstract

(1) Background: Neurological deficits associated with coronavirus disease (COVID-19) exacerbate respiratory dysfunction, necessitating rehabilitation strategies that address both. Previous studies have demonstrated that spinal cord transcutaneous stimulation (scTS) can facilitate the excitation of respiratory spinal neural networks in patients with post-COVID-19 syndrome. This study evaluates the efficacy of combining scTS with respiratory training (RT) to improve respiratory function in individuals with post-COVID-19 pulmonary deficits; (2) Methods: In this before–after, case-controlled clinical trial, five individuals with post-acute COVID-19 respiratory deficits participated in two interventional programs: 10 daily sessions of respiratory training (RT), followed by 10 daily sessions of scTS combined with RT (scTS + RT). Forced vital capacity (FVC), peak inspiratory flow (PIF), peak expiratory flow (PEF), time-to-peak inspiratory flow (tPIF), and time-to-peak expiratory flow (tPEF) were assessed at baseline and after each program; (3) Results: Compared to RT alone, the scTS + RT intervention resulted in an average effect size that was twice as large, with significant increases in FVC and PEF, and a significant decrease in tPEF; (4) Conclusions: The scTS-induced activation of respiratory neuronal networks, when combined with respiratory training, offers a promising therapeutic approach for treating persistent respiratory deficits in patients with post-acute COVID-19 syndrome.

## 1. Introduction

The Human Coronavirus Disease 2019 (COVID-19) is a systemic illness that exerts widespread effects on multiple physiological systems, affecting not only the respiratory system but also cardiovascular, neurological, and immune functions [1]. While COVID-19 is primarily known for its respiratory complications, its influence on other systems underscores its complex and multifaceted nature as a whole-body disease. Among its long-term impacts, post-acute COVID-19 syndrome has emerged as a particularly severe respiratory condition with extensive implications for healthcare systems, as the growing population of patients with persistent symptoms continues to demand significant medical and rehabilitative resources [2,3]. Although evidence indicates that the nervous system is also impacted by COVID-19, with many patients experiencing symptoms related to neuroinflammation and direct neural invasion, the respiratory and cardiovascular systems remain the primary targets of the virus [4]. These systems are the most frequently affected [3,5] and represent the most common causes of COVID-19-related morbidity and mortality [6], necessitating targeted strategies to manage and rehabilitate affected patients.

Although most patients recover after the acute phase of COVID-19, clinicians face significant challenges in managing the post-acute phase, which involves a broad spectrum of persistent symptoms that can affect multiple organ systems [7]. Respiratory symptoms associated with restrictive pulmonary pattern remain among the most prevalent and concerning [8,9,10]. In addition to respiratory complications, there is accumulating evidence that COVID-19 can also lead to various neurological symptoms, further complicating patient care and recovery pathways [4]. The mechanisms underlying these diverse complications are complex and multifactorial, involving direct viral invasion of neural tissue, as well as indirect effects such as immune-mediated responses, vascular damage, hypoxia, and systemic inflammation [11]. Therefore, effective rehabilitation for patients with post-acute COVID-19 must take a multifaceted approach that addresses these interconnected physiological challenges, tailoring interventions to manage respiratory, neurological, and other systemic deficits [9,12].

There has been a recent surge in research focusing on patient-specific rehabilitation programs aimed at enhancing respiratory outcomes for both acute and chronic COVID-19 patients. Personalized rehabilitation approaches hold promise, as they are designed to address the unique and varied needs of COVID-19 survivors. While respiratory rehabilitation has demonstrated clear effectiveness in managing many chronic respiratory disorders, its specific impact on COVID-19-related respiratory deficits remains uncertain, as the underlying pathology and recovery trajectory of post-COVID-19 respiratory impairment may differ from other conditions [13]. Current rehabilitation strategies for COVID-19 are predominantly centered on symptomatic relief, supported by multidisciplinary approaches that tailor treatment to individual patient needs [14]. To date, however, no definitive rehabilitation protocol has been established specifically for COVID-19, highlighting a critical gap in standard treatment approaches [7,15]. Most research efforts have prioritized acute care settings, with less attention given to the long-term rehabilitative needs of post-acute COVID-19 patient [16]. Consequently, the demand for comprehensive post-acute care has surged, with a marked increase in patients presenting to specialized healthcare facilities with diverse levels of respiratory dysfunction [17].

Previous research has demonstrated that respiratory training (RT) interventions can help improve respiratory deficits in patients with chronic respiratory disorders. RT aims to strengthen respiratory muscles, increase lung capacity, and improve overall functional outcomes, making it a valuable component of pulmonary rehabilitation [18,19,20,21,22]. However, while RT can provide measurable benefits, its effectiveness as a standalone intervention remains limited, particularly for patients with complex respiratory impairments like those associated with post-COVID-19 syndrome [23]. Given the risk of progressive deterioration in respiratory function, initiating respiratory rehabilitation during the acute phase of COVID-19 is generally not recommended due to potential complications [17], but becomes crucial in the post-acute phase to address the respiratory impairments associated with recovery from COVID-19 [24]. Recent studies suggest that neuromodulation approaches may offer a complementary benefit to traditional rehabilitation strategies, providing additional support for individuals recovering from the respiratory and neurological sequelae of COVID-19 [25,26]. Neuromodulation encompasses various techniques, including both invasive and non-invasive methods, that aim to stimulate specific neural circuits to promote neuroplasticity and functional recovery. Although several neuromodulatory techniques are available, few have been rigorously evaluated for their effectiveness in COVID-19 patients, leaving a gap in our understanding of how these approaches might enhance respiratory rehabilitation in this unique patient population [26,27].

In our recent study, we explored the application of a single session of spinal cord transcutaneous stimulation (scTS) over the thoracic spinal cord in post-COVID-19 patients, demonstrating improved respiratory performance likely due to activation of spinal neural networks involved in respiratory control [25]. These findings indicate that spinal cord stimulation, by enhancing neural responsiveness, could be a valuable adjunct to rehabilitation strategies for post-acute COVID-19 patients, supporting improved respiratory function and potentially accelerating recovery [13,25,26]. Based on this evidence, we hypothesized that combining scTS with RT would amplify use-dependent plasticity, resulting in significantly improved functional outcomes compared to RT alone. This combined approach could provide a more robust solution for addressing the complex respiratory challenges faced by individuals in post-acute recovery from COVID-19.

The objective of this study is to justify the development of a novel approach in respiratory rehabilitation by combining RT with scTS. This approach aims to assess respiratory functional responses to scTS in combination with RT, marking an important step toward our long-term goal of developing effective, evidence-based rehabilitation strategies for patients with COVID-19-induced respiratory deficits. Our central hypothesis is that scTS increases the excitability of motor networks responsible for respiration, thereby enhancing use-dependent neural plasticity in response to RT. The rationale for the proposed study lies in the critical need for innovative interventions to address persistent respiratory dysfunction in this population.

## 2. Materials and Methods

### 2.1. Research Participants

The study was approved by the Ethics Committee of Pavlov’s Institute of Physiology, St. Petersburg, Russian Federation (protocol #20-02, dated 18 December 2020), and conducted in accordance with the requirements of the Ministry of Science and Higher Education of the Russian Federation regarding “On the activities of organizations subordinate to the Ministry of Science and Higher Education of the Russian Federation in the conditions of preventing the spread of the COVID-19 infection in the territory of the Russian Federation” (order #692, dated 28 May 2020). Data collection took place in January and February 2021 in a physiological laboratory environment. Outcome measures were assessed before the initial intervention period (“pre-intervention” time point) and within the two-day window following each intervention: after 10 sessions of RT and after the completion of 10 sessions of RT combined with scTS (“post-intervention” time points).

Five participants (two females and three males), aged 56 ± 16 years, who had previously been hospitalized for severe COVID-19 with associated pneumonia, were recruited 57 ± 32 days after their initial diagnosis (Table 1). Prior to enrollment, all participants tested negative for COVID-19 via PCR. The inclusion criteria included a minimum age of 21 years, a diagnosis of post-acute COVID-19 syndrome, no ventilator dependence, a respiratory functional deficit defined by at least a 20% reduction in predicted FVC values, no history of tobacco or drug use, and the absence of cardiovascular or respiratory conditions unrelated to COVID-19. At the time of the study, all participants reported experiencing episodes of shortness of breath and fatigue. Throughout the study, participants maintained their regular daily activities, and none withdrew from the study.

### 2.2. Research Procedures

The study included one cohort of participants (*n* = 5) who underwent two different interventions: RT alone and RT combined with scTS. Two datasets associated with RT alone and RT + scTS, have been directly compared to evaluate differences between these conditions. During the interventional sessions, participants were seated in a chair with an approximately 45° head-up tilt.

#### 2.2.1. Respiratory Training (RT)

RT was delivered using a threshold Positive Expiratory Pressure (PEP, Philips Respironics Inc., Cedar Grove, NJ, USA) device. Over two weeks, participants completed ten 30-min sessions, performing expiratory efforts against a pressure threshold load until their lungs felt empty. The RT program began with a pressure threshold of 7 cm H_2_O, progressively increasing to 13 cm H_2_O by session 10, as tolerated. The interval training protocol consisted of five work sets, each lasting 3 min, with rest intervals of 1 to 3 min between sets.

#### 2.2.2. Spinal Cord Transcutaneous Stimulation (scTS)

Following the post-RT assessments, participants underwent an additional 10 sessions of scTS combined with RT. During each session, scTS was administered first, followed by the RT protocol as described above. Multi-site scTS was delivered using a Neostim-5 device (Cosyma Inc., Denver, CO, USA). Stimulation was applied to the midline between the third and fourth, as well as between the eighth and ninth thoracic spinous processes (Th3–Th4 and Th8–Th9), corresponding to the T5 and T10 spinal cord segments, respectively [28]. Self-adhesive cathode electrodes, 32 mm in diameter (ValueTrode, Axelgaard Manufacturing Co., Ltd., Fallbrook, CA, USA), were used for electrode placement [28]. Two interconnected self-adhesive rectangular electrodes, each measuring 5 × 9 cm, were used as anodes and positioned bilaterally along the rectus abdominis muscles, centered at the level of the umbilicus.

**Figure 1:**
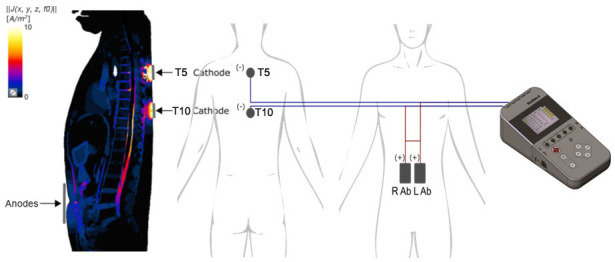
Placement of the scTS electrodes and sagittal view of the current field density using computational modeling carried out ohmic quasi-static simulations (Sim4Life, Zurich Med Tech, Switzerland) in a virtual model (Yoon Sun ViP 4.0, IT’IS Foundation) with tissue-specific electrical properties. Note that cathodes (−) are positioned on the skin between the Th3–Th4 and Th8–Th9 spinous processes, aligning with the T5 and T10 spinal cord segments, respectively; the anodes (+) are located over the abdomen (Ab), horizontally centered at the level of the umbilicus.

To identify the primary areas targeted during scTS, simulations of current field visualization were conducted to clarify spinal structures engaged by scTS at each stimulation site. These simulations used an ohmic quasi-static model, employing a finite element approach (Sim4Life, Zurich Med Tech, Zurich, Switzerland) within a virtual framework (Yoon Sun ViP 4.0, IT’IS Foundation) incorporating tissue-specific electrical properties [29]. The current amplitudes in the simulation mirrored the average values applied in the interventional settings (Figure 1).

The stimulation protocol involved 5 kHz-modulated monophasic pulses with a 1 ms duration, delivered at a frequency of 30 Hz to target spinal cord segments associated with accessory respiratory muscles. The stimulation intensity was initially set at 10 mA and gradually increased to 85 mA, until motor threshold was indicated by intercostal or abdominal muscle twitching. During the intervention, the intensity was reduced by 10 mA to sub-motor threshold levels and applied for 20 min. Brachial arterial blood pressure (BP), heart rate (HR), oxygen saturation, and pain perception (measured using a visual analog scale of pain, VASp) were continuously monitored 20 min before, during, and 20 min after each intervention. All scTS sessions were conducted by qualified technologists.

Textual editing for clarity and grammar of the manuscript was performed with the aid of an AI language model (ChatGPT, OpenAI, San Francisco, CA, USA). The AI provided recommendations on sentence structure, phrasing, and grammar to enhance readability and ensure precise scientific communication. No changes were made to citations, data interpretation, or scientific content [30].

### 2.3. Outcome Measures

Respiratory function outcomes, including forced vital capacity (FVC, L and % predicted), peak inspiratory flow (PIF, L/min), peak expiratory flow (PEF, L/min), time-to-peak inspiratory flow (tPIF, sec), and time-to-peak expiratory flow (tPEF, sec), were assessed using flow-volume curves recorded in the seated position during standard spirometry. These measurements were collected using a Powerlab 16/35 data acquisition system and the Human Respiratory Kit (AD Instruments, Denver, CO, USA) [31]. FVC served as a reliable endpoint for evaluating the effectiveness of respiratory interventions [32,33].

### 2.4. Statistical Analysis

The power analysis for this study was performed to ensure sufficient power (over 80%) to detect the scTS effect using data from our previous pilot study [25]. This study, conducted with 10 participants, revealed a pre-post scTS change in tPEF with an effect size (Cohen’s d) equal to 0.77, classified as medium by Sawilowsky’s [34] extended criteria [35]. To reliably detect this effect size (ES = 0.77) with at least 80% power using a 2-sided paired t-test with a significance level of 5%, a sample size of 5 participants is required. We adjusted for multiple testing using the Bonferroni correction and accounted for potential data loss by planning to enroll 7 participants.

Outcomes that were normally distributed, as determined by the Shapiro–Wilk test, are summarized using the mean and standard deviation (SD) and evaluated with a paired *t*-test. Non-normally distributed outcomes are summarized with the median, interquartile range (25th–75th percentiles), and median absolute deviation (MAD), and analyzed using the Signed Rank test. All tests were two-sided, with a significance level set at 5%. Pre-post changes were quantified using “Cohen’s d” effect size [36], calculated as the mean of the differences divided by the SD of differences for normally distributed outcomes, and as the median of differences divided by the MAD of differences for non-normally distributed outcomes. Effect sizes were classified according to Sawilowsky’s extension [34] of Cohen’s criteria as trivial (<0.01), very small (0.01–0.19), small (0.2–0.49), medium (0.5–0.79), large (0.8–1.19), very large (1.2–1.99), and huge (≥2.0). A threshold of 0.5 was considered the minimally important difference for health-related quality of life [37,38]. The statistical significance threshold was set to α = 0.05.

## 3. Results

**Figure 2:**
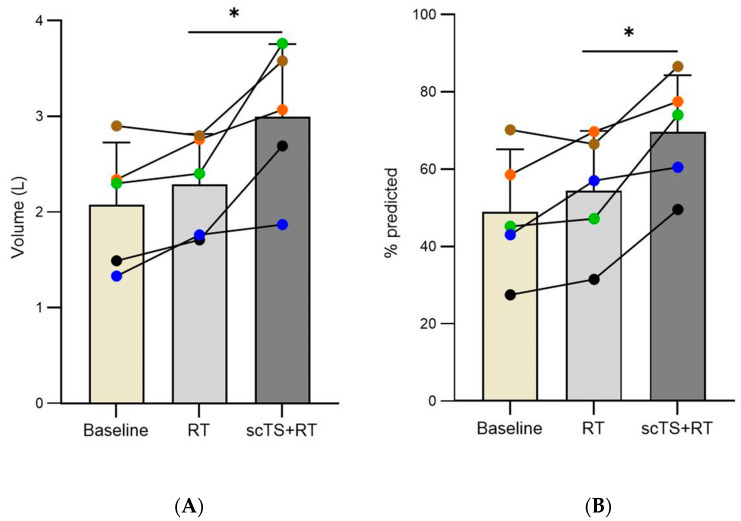
(**A**) Forced volume capacity (FVC) expressed in actual values (L) or (**B**) as a percent of predicted values (% predicted) at baseline, after RT, and after scTS + RT in post-acute COVID-19 individuals (*n* = 5). Note the linked points representing the changes in a particular individual before and after intervention. * Denotes significant difference.

Compared to baseline, the RT intervention no alteration was observed in FVC changed from 2.07 ± 0.65 to 2.28 ± 0.52 L (*p* = 0.131) and from 48.9 ± 16.23 to 54.38 ± 15.52% predicted (*p* = 0.1603). Following the scTS + RT intervention, FVC outcomes significantly increased compared to post-RT levels, rising from 2.28 ± 0.52 to 2.99 ± 0.76 L (*p* = 0.034) and from 54.38 ± 15.52 to 69.64 ± 14.61% predicted (*p* = 0.023) (Figure 2).

**Figure 3:**
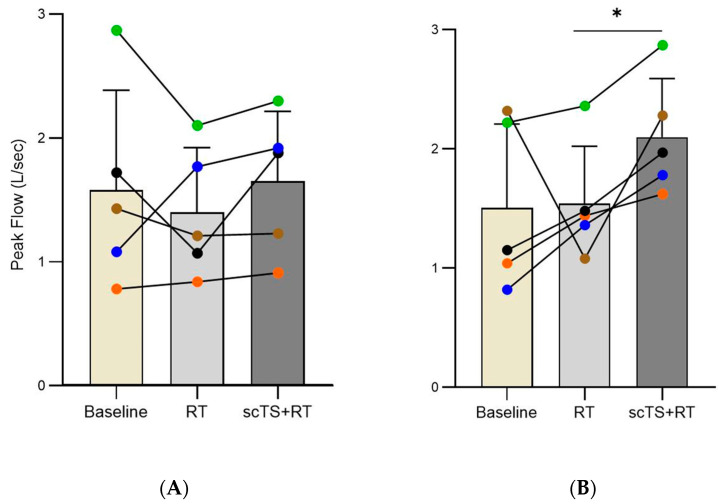
(**A**) Peak inspiratory flow (PIF) and (**B**) peak expiratory flow (PEF) at baseline, after RT, and after scTS + RT in post-acute COVID-19 individuals (*n* = 5). Note the linked points representing the changes in a particular individual before and after intervention. * Denotes significant difference.

After the RT intervention, there was no significant difference in PIF changed from 1.58 ± 0.80 to 1.40 ± 0.52 L/s (*p* = 0.536). Following the scTS + RT intervention, PIF did not significantly changed as well: from 1.21 ± 0.37 to 1.88 ± 0.42 L/s (Median ± MAD, *p* = 0.063) (Figure 3A). PEF changed from 1.15 ± 0.37 to 1.38 ± 0.04 L/s (Median ± MAD, *p* = 0.625) after RT and significantly increased from 1.52 ± 0.49 to 2.11 ± 0.49 L/s (*p* = 0.023) after the scTS + RT intervention (Figure 3B).

**Figure 4:**
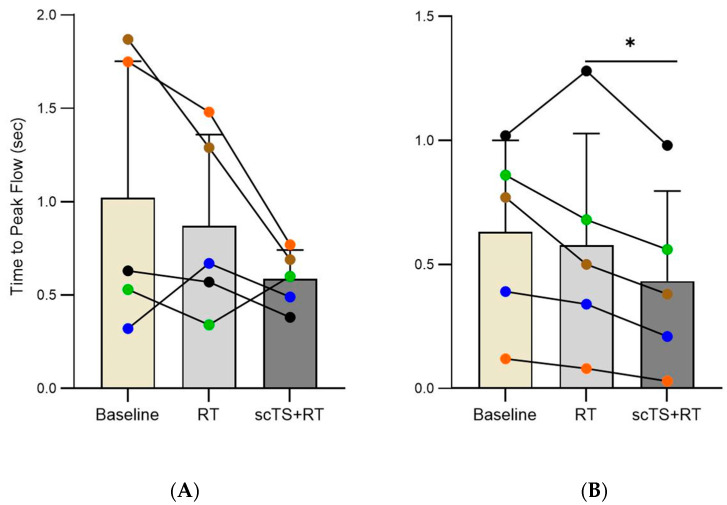
(**A**) Time to peak of inspiratory flow (tPIF) and (**B**) time to peak of expiratory flow (tPEF) at baseline, after RT, and after scTS + RT in post-acute COVID-19 individuals (*n* = 5). Note the linked points representing the changes in a particular individual before and after intervention. * Denotes significant difference.

**Figure 5:**
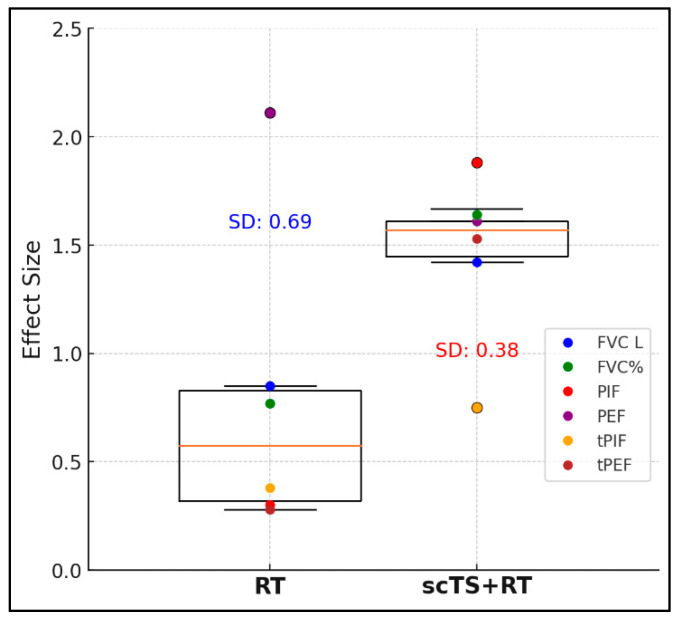
Effect size of overall FVC, PIF, PEF, tPIF, and tPEF changes after RT alone and in combination with scTS (*n* = 5).

The RT intervention changed tPIF from 1.00 ± 0.75 to 0.87 ± 0.49 sec (*p* = 0.444) and tPEF from 0.63 ± 0.37 to 0.56 ± 0.46 sec (*p* = 0.506). The scTS + RT intervention changed tPIF from 0.87 ± 0.49 to 0.58 ± 0.16 sec (*p* = 0.171) and led to a significant decrease in tPEF from 0.56 ± 0.46 to 0.43 ± 0.36 sec (*p* = 0.013) (Figure 4). The average effect sizes of the RT and scTS + RT interventions accounted for all outcome measures were 0.78 ± 0.70 vs. 1.46 ± 0.38 representing “medium” vs. “very large” effect sizes, respectively (Figure 5).

## 4. Discussion

This study represents the first investigation into the effectiveness of combining RT with scTS as a neuromodulatory strategy for respiratory rehabilitation in patients affected by post-COVID-19 pulmonary impairments. While RT alone yielded only modest improvements in respiratory function, the integration of scTS with RT resulted in notably enhanced therapeutic outcomes. Specifically, the average effect size for the combined intervention was approximately twice as large as that observed with RT alone, underscoring the synergistic benefit of this novel approach. Our findings indicate that scTS-induced activation of respiratory neuronal networks can serve as an effective neuromodulatory method for restoring respiratory function in patients with post-COVID-19 pulmonary deficits. The mechanism appears to involve targeted stimulation of respiratory-related neural pathways, which may support neural plasticity and functional recovery of compromised respiratory control systems. This approach highlights the potential of scTS to directly augment neural network responsiveness, thereby facilitating improved respiratory outcomes when coupled with RT. Considering the substantial number of COVID-19 patients in need of post-acute respiratory rehabilitation, neuromodulatory strategies like scTS could be valuable adjunctive therapies for clinicians. Such interventions could complement existing rehabilitation protocols and other therapeutic modalities to improve respiratory function, quality of life, and overall health outcomes for individuals recovering from COVID-19-related respiratory issues. Integrating neuromodulation as part of a multidisciplinary rehabilitation plan may offer a promising path to address the growing demand for effective post-COVID-19 care solutions [10].

Recent research has increasingly focused on the development of patient-specific rehabilitation programs tailored to the unique needs of individuals recovering from COVID-19. These personalized approaches have demonstrated potential in improving respiratory outcomes for both acute and chronic COVID-19 patients, allowing for more targeted and effective interventions that address each patient’s specific deficits and recovery trajectory [13]. Existing studies highlight significant uncertainties in quantifying the effectiveness of technology-based cardiopulmonary training programs and emphasize the need for patient-preference research, including the choice between remote rehabilitation and technology-driven programs in specialized centers [39]. Other studies suggest that combining active physiotherapy with pharmacological treatment significantly alleviates symptoms such as dry cough and dyspnea in COVID-19 patients, with symptom improvement varying by the type of physiotherapy applied [40]. In line with this trend, our study explored the effects of RT alone, and our findings are consistent with existing literature showing that RT can enhance respiratory muscle strength and functional capacity in individuals with Long COVID-19, potentially leading to improved endurance and breathing efficiency. However, studies have also indicated that RT alone is limited in its capacity to improve actual lung function, suggesting a need for additional therapeutic approaches to optimize respiratory rehabilitation [27]. Our objective, therefore, was to build on the foundational benefits of RT by introducing a neuromodulatory component to enhance its effectiveness. Previously, we demonstrated that scTS, when applied over the thoracic spinal cord in an acute setting, significantly enhances the activity of spinal neural networks involved in respiratory motor control [25]. This initial finding pointed to scTS as a promising tool for amplifying neural network responsiveness linked to respiratory function. In our current study, we expanded upon these results, showing that repeated applications of scTS, when integrated with traditional respiratory rehabilitation approach, lead to substantially improved therapeutic outcomes in patients with post-COVID-19 syndrome. This combined approach appears to strengthen the activation of respiratory-related neural pathways, thereby improving both functional recovery and respiratory efficiency. In addition to scTS, recent research has highlighted several other neuromodulatory techniques that could serve as complementary interventions in the rehabilitation of COVID-19 patients. For example, electrical stimulation of cranial nerves and transcranial magnetic stimulation have been explored as promising methods for augmenting therapeutic effects, especially during the acute phase of COVID-19. These techniques, which target various aspects of neural network responsiveness, may help to reduce the severity of respiratory impairments associated with COVID-19 and support the recovery process in a comprehensive manner [26]. Altogether, these neuromodulatory approaches represent a new frontier in respiratory rehabilitation, offering clinicians a broader array of tools to address the complex respiratory challenges faced by COVID-19 patients. Integrating these strategies with traditional rehabilitation protocols may ultimately enhance recovery and improve the quality of life for individuals affected by long-term COVID-19 respiratory issues.

The findings of this study present notable clinical implications for addressing respiratory deficits in post-COVID-19 patients, particularly by the neuromodulatory techniques. Neuromodulation, when combined with traditional respiratory muscle training, appears to provide additive—or potentially exponential—benefits, enhancing respiratory outcomes beyond those typically achieved through conventional therapy alone. The neuromodulatory approach used in this study led to significant improvements in key respiratory metrics, including forced vital capacity (FVC), peak expiratory flow (PEF), and time to peak expiratory flow (tPEF). These improvements may translate into meaningful clinical benefits, such as symptom alleviation, improved respiratory function, and potentially reduced mortality. According to the European Respiratory Society’s standards for pulmonary function testing, an increase of 10% in FVC is recognized as a significant bronchodilator response [33]. Similarly, pulmonary rehabilitation has long been associated with reduced mortality in respiratory patients [41], and has demonstrated an FVC improvement of 7.1% in patients who completed a pulmonary rehabilitation program after mechanical ventilation for severe COVID-19 pneumonia [42]. In the context of our study, the significant gains in peak flow measurements and shortened time to reach peak flow with scTS may indicate enhanced engagement of accessory respiratory muscles and more efficient respiratory muscle recruitment. These improvements, which serve as indirect indicators of heightened respiratory neuromuscular activity, likely contribute to enhanced expiratory function, stronger cough ability, and improved airway clearance [43]. The results from this study suggest that neuromodulation combined with respiratory muscle training could yield clinical outcomes comparable to those observed with bronchodilators or traditional pulmonary rehabilitation programs. This combination approach may facilitate improvements in respiratory function, enabling patients to achieve better pulmonary health and overall quality of life. Furthermore, the timing of intervention could be a critical factor in maximizing recovery outcomes. Early activity-based interventions administered within intensive care units (ICUs) have demonstrated benefits in mitigating functional impairments, increasing ventilator-free days, enhancing patients’ quality of life, and reducing the risk of post-discharge mortality [44,45]. These findings underscore the importance of incorporating early neuromodulatory strategies—potentially in combination with pulmonary rehabilitation—within clinical protocols to enhance respiratory recovery in post-COVID-19 patients.

Integrating these approaches into rehabilitation practices could have substantial implications for clinical care, offering clinicians an expanded toolkit to address the multifaceted respiratory complications associated with COVID-19. Early intervention with neuromodulation may provide a proactive approach to counteract respiratory muscle deconditioning and promote faster recovery, supporting both acute and long-term functional outcomes. As our understanding of the benefits of neuromodulatory strategies continues to evolve, these interventions could play an increasingly vital role in the rehabilitation and long-term care of individuals with post-COVID-19 respiratory deficits.

### Limitations and Future Directions

Several limitations of this study should be acknowledged, and they highlight key areas for future research. First, while the initial results suggest that scTS can be effective in respiratory rehabilitation, the study did not include a test–retest methodology or matched control groups, which are essential for establishing more robust conclusions. The study’s small sample size and lack of a control group limit the scope for robust conclusions. However, this study was designed as a preliminary investigation to generate data for a future, adequately powered clinical trial. Future large-scale clinical trials should incorporate rigorous experimental designs with appropriate control groups, such as those receiving sham stimulation or non-targeted scTS configurations, to better isolate the specific neuromodulatory effects of scTS on respiratory outcomes. These controls will help ensure that any observed benefits are directly attributable to scTS targeting respiratory motor networks.

Second, while the therapeutic potential of scTS was demonstrated, there is room for optimizing the stimulation protocol. Future research should investigate variations in electrode positioning and stimulation parameters to better target respiratory motor networks. Computational modeling and functional studies could provide valuable insights into how changes in current intensity, frequency, and electrode placement impact the specificity and effectiveness of scTS. By refining these parameters, the therapeutic efficacy of scTS could be significantly enhanced.

Additionally, the current study employed a sequential application of scTS followed by RT, but it remains uncertain whether this sequence is superior to a concurrent approach where scTS and RT are administered simultaneously. The timing of neuromodulation in relation to motor training may be a critical factor in determining therapeutic outcomes. Experimental validation of these different approaches is needed to better understand how the timing of interventions influences respiratory network activation and rehabilitation success.

Furthermore, while this study focused on post-acute COVID-19 patients, it is important to explore the effectiveness of the scTS + RT intervention in other patient populations with respiratory deficits, such as individuals with chronic respiratory conditions or those with neurological impairments. Broadening the scope of research to include these groups will determine whether the combined intervention has universal applicability across various conditions.

Finally, the long-term sustainability of the improvements achieved through scTS + RT needs further investigation. Future studies should assess how frequently patients need stimulation sessions to maintain therapeutic gains and whether the benefits persist after the intervention is discontinued. Understanding the durability of the intervention’s effects will be essential for determining its practical application in clinical settings.

## 5. Conclusions

1. This study demonstrates that combining scTS with RT offers significant benefits for respiratory rehabilitation in post-COVID-19 patients. The neuromodulatory effects of scTS led to substantial improvements in respiratory function, particularly in expiratory motor networks, with an average effect size twice that of RT alone. These findings suggest that scTS could enhance recovery, especially in patients with severe expiratory muscle weakness, offering clinical benefits comparable to or exceeding traditional rehabilitation methods.
2. Given the increasing emphasis on personalized rehabilitation, the ability to tailor scTS to individual patient needs highlights its potential for more targeted and effective treatment. Additionally, the broader application of neuromodulation, including scTS, to other neurological and chronic respiratory conditions supports its relevance as a key component in respiratory rehabilitation programs.
3. Future research should focus on optimizing scTS protocols, exploring the ideal timing and application in combination with RT, and expanding its validation across a wider range of patient populations with respiratory and neurological deficits. Incorporating scTS into standard rehabilitation protocols could significantly improve outcomes for patients with long-term respiratory impairments, both in post-COVID-19 and other respiratory conditions.

## Figures and Tables

**Table 1:** Demographic summary of participants.

ID	Age (year)	Sex	Height (cm)	Weight (kg)	BMI (kg/m^2^)	FVC (% pred.)
05K	47	F	170	62	21.45	70
09K	59	M	182	110	33.21	66
10K	34	M	178	88	27.77	71
11K	64	M	170	75	25.95	65
13K	76	F	169	71	24.86	65
Mean ± SD	56 ± 16	NA	174 ± 6	81 ± 19	27 ± 4	67 ± 3

Abbreviations: BMI, body mass index; F, female; FVC, forced vital capacity; ID, identification; M, male; NA, not applicable; pred., predicted.

## Data Availability

The data supporting the findings of this study are available upon request from the corresponding author.
